# EpCAM-positive circulating tumor cells and serum AFP levels predict outcome after curative resection of hepatocellular carcinoma

**DOI:** 10.1038/s41598-023-47580-0

**Published:** 2023-11-27

**Authors:** Lorenz Kocheise, Martin Schoenlein, Berit Behrends, Vincent Joerg, Christian Casar, Thorben W. Fruendt, Thomas Renné, Asmus Heumann, Jun Li, Samuel Huber, Ansgar W. Lohse, Klaus Pantel, Sabine Riethdorf, Henning Wege, Kornelius Schulze, Johann von Felden

**Affiliations:** 1 I. Department of Medicine, University Medical Center Hamburg-Eppendorf, Martinistr. 52, 20246 Hamburg, Germany; 2 Department of Oncology, Hematology and Bone Marrow Transplantation with Section Pneumology, Hubertus Wald University Comprehensive Cancer Center Hamburg, University Medical Center Hamburg-Eppendorf, Hamburg, Germany; 3 Institute of Clinical Chemistry and Laboratory Medicine, University Medical Center Hamburg-Eppendorf, Hamburg, Germany; 4 Bioinformatics Core, University Medical Center Hamburg-Eppendorf, Hamburg, Germany; 5 Irish Centre for Vascular Biology, School of Pharmacy and Biomolecular Sciences, Royal College of Surgeons in Ireland, Dublin, Ireland; 6 Center for Thrombosis and Hemostasis (CTH), Johannes Gutenberg University Medical Center, Mainz, Germany; 7 Department of General, Visceral and Thoracic Surgery, University Medical Center Hamburg-Eppendorf, Hamburg, Germany; 8 Department of Tumor Biology, University Medical Center Hamburg-Eppendorf, Hamburg, Germany; 9 Cancer Center Esslingen, Klinikum Esslingen, Esslingen, Germany

**Keywords:** Tumour biomarkers, Surgical oncology

## Abstract

Hepatocellular carcinoma (HCC) has high recurrence rates exceeding 50% despite curative resection. The serum biomarker alpha-fetoprotein (AFP) is a well-known prognostic marker for HCC. EpCAM-positive circulating tumor cells (CTC) have a high predictive value for early HCC recurrence after curatively intended resection, most likely indicating micro-metastases at the time of resection. However, sensitivity remains low. The objective of this study was to evaluate a composite test comprising both CTC and AFP to identify patients at high risk for early HCC recurrence. We prospectively enrolled 58 patients undergoing curative intended resection for HCC at a tertiary referral center. Blood specimens were obtained prior to resection and analyzed for EpCAM-positive CTC and serum AFP levels. A positive result was defined as either detection of CTC or AFP levels ≥ 400 ng/ml. Eight patients tested positive for CTC, seven for AFP, and two for both markers. A positive composite test was significantly associated with shorter early recurrence-free survival (5 vs. 16 months, *p* = 0.005), time to recurrence (5 vs. 16 months, *p* = 0.011), and overall survival (37 vs. not reached, *p* = 0.034). Combining CTC and AFP identified patients with poor outcome after surgical resection, for whom adjuvant or neoadjuvant therapies may be particularly desirable.

## Introduction

Liver cancer is one of the most prevalent cancers worldwide, with an estimated number of more than 900,000 new cases per year. Among these cases, hepatocellular carcinoma (HCC) accounts for more than 90% of all cases^1^. Despite therapeutic advances in recent years, the overall prognosis is still poor. Curative treatment is limited to early disease stages using surgical resection, local ablation procedures, or liver transplantation. Surgical resection is complicated in many cases by the underlying liver disease, in particular liver cirrhosis and associated with significant risks for the patient. Even after successful surgical resection HCC recurrence rates exceed 50%. In clinical practice, a cut-off of 24 months was established to distinguish between early and late recurrence. In other words, recurrence due to progressive intrahepatic micro-metastases or de novo tumor formation in the preneoplastic cirrhotic liver^2,3^. Prognostic markers that reliably predict early recurrence after surgical resection are of great interest to identify patient groups that might not benefit from tumor surgery alone. With the advent of effective immunotherapy^4,5^ identification of patients at high risk for recurrence seems of additional interest. These patients might benefit from neoadjuvant or adjuvant therapy.

Several biomarkers have been proposed to predict a high risk of recurrence after surgical resection or ablation of the primary cancer site. Alpha-fetoprotein (AFP) as the most common HCC biomarker has been particularly thoroughly investigated in this regard. Previous studies have shown that patients with preoperative AFP levels greater than 400 ng/ml have higher rates of recurrence and shorter recurrence-free survival than patients with AFP levels below 400 ng/ml^6–8^ after surgical resection. However, AFP is only elevated in less than half of all patients with HCC^9^. Alternative biomarkers include des-γ-carboxy prothrombin (DCP) and lens culinaris agglutinin-reactive fraction of α-fetoprotein (AFP-L3). Both have been studied particularly in the context of HCC surveillance in patients at risk^10^. Limited data suggest that elevated AFP-L3 and DCP levels are associated with worse recurrence-free survival, albeit with small observed effect sizes^11–14^.

Detection of micro-metastases or surrogates of systemic tumor manifestation, e.g. circulating EpCAM-positive tumor cells (CTC), is considered to indicate more advanced disease states with worse prognosis. The presence of EpCAM-positive CTC has been shown to be essential to the process of cancer metastasis. These neoplastic cells are released from the primary tumor into the bloodstream after epithelial-mesenchymal transition (EMT) initiating metastatic growth at distant sites^15^. As previously shown by our and other groups, detection of EpCAM-positive CTC in peripheral blood prior to surgery correlates with recurrence and recurrence-free survival after resection or liver transplantation^16–20^. However, while CTC have a high predictive value for early HCC recurrence after curative resection, sensitivity remains low.

Although several biomarkers have already been shown to be specific for recurrence prediction, the sensitivity of each biomarker was insufficient. The purpose of this study was to investigate the benefit of combining AFP levels and the detection of CTC to allow an increase in sensitivity without loss of specificity.

## Patients and methods

### Patients

For this study, 58 patients diagnosed with HCC who underwent liver resection between July 2011 and October 2015 at the University Medical Center Hamburg-Eppendorf were consecutively enrolled and prospectively followed up. Only patients with histologically confirmed HCC were included. Recurrence was diagnosed applying current imaging guidelines (mRECIST) as proposed by the European Association for the Study of the Liver (EASL)^21^ and confirmation by biopsy in case of inconclusive contrast dynamics was performed. Two patients had to be excluded because of death or liver transplantation within 30 days after resection due to acute liver failure after surgery. Hence, the overall survival analysis was performed on 56 patients. Of these, 49 patients had available follow-up imagining and were eligible for recurrence analysis. All patients were followed up until death or effective time of data analysis in May 2020. Parts of this cohort have previously been reported^16^.

### Clinical information

Clinical characteristics such as demographic data, risk factors, underlying chronic liver disease or cirrhosis, tumor stage according to the BCLC staging system^22^, and presence of macroscopic or microscopic vascular invasion were recorded at time of liver resection. Data was obtained by review of medical records, including preoperative imaging, and/or surgical and pathological reports following resection. After liver resection, surveillance of patients was performed with regular cross-sectional imaging of the liver, clinical examination, and laboratory testing every three months according to current clinical guidelines^21^. All patients were followed up until death or effective time of data analysis in May 2020.

### Patients’ blood samples

Blood specimens (7.5 ml) were drawn within 24 h before surgical resection into CellSave™ Preservative Tubes (Menarini Silicon Biosystems, Bologna, Italy), stored at room temperature and processed within 96 h after collection. To avoid possible contamination with epithelial cells of the skin, one extra tube was filled prior to the assay tube. Serum samples were collected and stored at − 80 °C until further analysis for AFP, AFP-L3 and DCP was performed.

### CellSearch™ system (CSS)

The CSS is a semi-automated device detecting and enumerating EpCAM-positive/cytokeratin-positive CTC. This method was cleared by the FDA for patients with metastatic breast, prostate and colorectal cancer^23,24^. Further studies have successfully validated CSS for CTC detection in HCC^25,26^.The assay was performed according to manufacturer's instructions as described elsewhere^24^. Briefly, 7.5 mL of blood were mixed with 6 mL of buffer, centrifuged at 800 × g for 10 min, and then placed on the CellPrep system. First, the automated CelltracksTM AutoPrep system enriches cells with ferrofluid-coated anti-EpCAM-antibodies. Next, these cells are immunostained with fluorescently-labeled anti-keratin-antibodies identifying, among others, cytokeratins (CK) 8, 18, and 19. Nuclear staining with 4′,6-diamidino-2-phenylindole (DAPI) ensures integrity of nuclei, and anti-CD45-antibodies distinguish epithelial cells from leukocytes. After automated immunomagnetic enrichment and immunocytochemical staining cells were automatically scanned by the CellTracks Analyzer II (Menarini). Finally, image galleries were manually evaluated for CTC and keratin-, DAPI (4′,6-diamidino-2 phenylindole)-positive/CD45-negative cells with a diameter of at least 4 μm were referred to as CTC.

### AFP, AFP-L3, DCP analysis

AFP, ADP fucosylated isoform AFP-L3 and des-gamma-carboxy-prothrombin (DCP) were measured using the Micro Total Immunoassay System “µTASWako™ i30” (FUJIFILM Wako Diagnostics U.S.A. Corporation) according to the manufacturer's instructions.

### Statistical analysis

The primary endpoint of the study was investigator-assessed early recurrence-free survival (eRFS) within 24 months after resection using modified RECIST (mRECIST) criteria for tumor response^27^. Secondary endpoints included time to recurrence (TTR) within 24 months and overall survival (OS). Patients who underwent liver transplantation for HCC recurrence were censored at the time of liver transplantation. We used Fisher’s exact test, Pearson’s Chi Square test, and Student’s t-test to compare differences between categorical and continuous variables, respectively. For Cox regression modeling of non-binary coded parameters, e.g. tumor size (T status), and microvascular invasion (V status), a dichotomous fashion was used (e.g., T1/2 vs. T3, V0 vs. V1/2). Thirty-four eRFS events occurred. Multivariate Cox regression and adjustment was performed for all biomarkers, irrespective of *p*-values in the univariate analysis for the risk factors age, gender and BCLC stage. CTC analysis was nonparametric according to the existence of CTC (CTC negative vs. CTC positive). eRFS, TTR and OS were analyzed using log-rank test (Mantel–Haenszel Version) and plotted with Kaplan Meier curves. Sensitivity and specificity for recurrence prediction were calculated using CTC status and AFP serum levels. For all statistical analyses *p*-values below 0.05 were considered significant. All statistical analyses were conducted on GraphPad Prism (Version 10.0.3).

### Ethics declarations

This study was approved by the Ethics Committee of the Hamburg Medical Association (# PV3578) and written informed consent to the study protocol was obtained from all participants prior to inclusion in this study. All experiments were carried out in accordance with the relevant guidelines and regulations. All research was conducted in accordance with the Declaration of Helsinki. This study was not registered in a clinical trial registry.

## Results

### Patient characteristics

**Figure 1:**
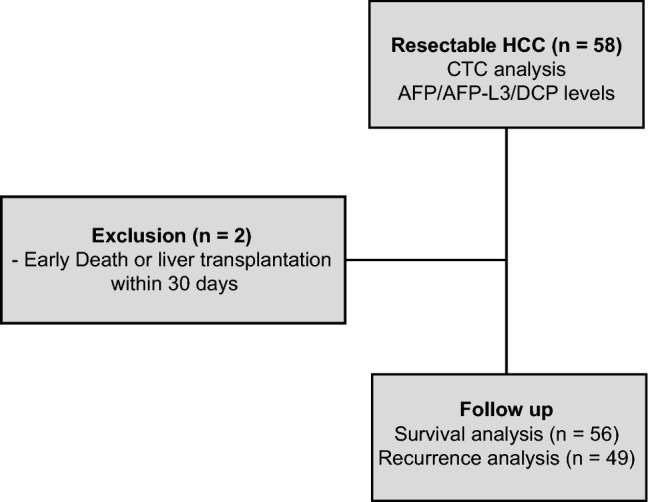
Study design. Flowchart showing the number of patients included or excluded at each stage of the analysis.

Patient selection is summarized in Fig. 1. In total, 58 patients were included, baseline characteristics are presented in Table 1. This patient cohort consisted of 49 (84.5%) men and 9 (15.5%) women. The mean age of the patients was 64.8 years. 27 (46.6%) of patients had confirmed cirrhosis at the time of HCC diagnosis. Etiology of the underlying liver disease was distributed as follows: Chronic viral hepatitis (n = 10 chronic hepatitis B, n = 15 chronic hepatitis C), non-alcoholic steatohepatitis (n = 10), chronic alcohol abuse (n = 14), autoimmune liver disease (n = 2), hemochromatosis (n = 1), cryptogenic (n = 6). 7 patients had more than one risk factor for HCC development. For patients with multiple risk factors, each risk factor was included. 7 patients had no detectable underlying liver disease. 44 (75.9%) of the HCCs were classified as BCLC stage A, while 14 (24.1%) were classified as BCLC stage B. All HCCs were considered suitable for curative resection by an interdisciplinary tumor board. Histological workup showed no vascular invasion in 35 (60.3%) of the resected specimens. 22 (37.9%) of the resected specimens showed vascular invasion, while for one resected specimen no statement regarding vascular invasion was made by the pathologist. Overall, our cohort reflects a typical cohort for HCC-resection in Western tertiary centers.

**Table 1:** Demographic characteristics of patients with resection of HCC and Biomarker analysis.

Baseline characteristics	Patients, n = 58 (%)
Gender (%)	
Male	49 (84.5)
Female	9 (15.5)
Age	
Mean ± SD	64.8 ± 10.6
Liver cirrhosis (%)	
Yes	27 (46.6)
No	31 (53.4)
Child–Pugh stage (%)	
A	25 (92.6)
B	2 (7.4)
Etiology of liver disease#	
Hepatitis B	10
Hepatitis C	15
NASH	10
Alcohol	14
Autoimmune liver disease	2
Hemochromatosis	1
Cryptogenic	6
No liver disease	7
BCLC staging (%)	
A	44 (75.9)
B	14 (24.1)
Vascular invasion (%)	
V0	35 (60.3)
V1/V2	22 (37.9)
No data available	1 (1.7)
Residual tumor (%)	
R0	54 (93.1)
R1	4 (6.9)
Recurrence (%)	
Early recurrence (< 24 months)	33 (56.9)
Late recurrence (> 24 months)	4 (6.9)
No recurrence	12 (20.7)
No data available	9 (15.5)

*SD* standard deviation, *V* vascular invasion, *R* resection margin.

^#^Multiple risk factors were possible (n = 65).

### Biomarker analysis for early recurrence-free survival

Consistent with the literature, surveillance imaging detected recurrence of HCC in 33 (56.9%) patients within the first 24 months. Four (6.9%) patients developed HCC recurrence after > 24 months, of which all patients had previously been diagnosed with liver cirrhosis. In accordance with standard clinical practice, these HCCs were therefore considered de novo tumors. Multiple biomarkers such as CTC, AFP, AFP-L3, and DCP were measured preoperatively in the patient's peripheral blood within 24 hours of resection. In the patient cohort with available recurrence data (n = 49), EpCAM-positive CTC were detected in 8 patients. Biomarkers above the upper limit of normal (ULN) were detectable for 24 (49.0%, AFP), 19 (38.8%, AFP-L3) and 18 (36.7%, DCP) patients respectively. AFP levels ≥ 400 ng/ml were detected in 7 (14.3%) patients. The effect of individual biomarkers, tumor characteristics, or patient characteristics on eRFS was examined using univariate Cox regression. Results are reported in Table 2 with corresponding hazard ratios (HR) and 95% confidence intervals (95% CI). There was no significant correlation between eRFS and baseline characteristics such as gender, age, liver cirrhosis, Child–Pugh stage, or BCLC stage. Incomplete resection status (R1) in histological examination of the resected tumor was associated with worse eRFS (HR 3.0, 95% CI 0.9–8.3, *p* = 0.047). A trend toward worse eRFS was also evident for vascular invasion, although not statistically significant (HR 1.8, 95% CI 0.9–3.6, *p* = 0.098).

**Table 2:** Cox proportional hazards regression analysis for early recurrence free survival (eRFS).

Risk factors	HR (95% CI)	*p*-value		
Gender	1.6 (0.6–3.6)	0.320		
Age	1.0 (1.0–1.0)	0.145		
Liver cirrhosis	0.8 (0.4–1.6)	0.565		
Child–Pugh stage	4.0 (0.6–17.1)	0.083		
BCLC staging	1.3 (0.5–2.7)	0.531		
Vascular invasion	1.8 (0.9–3.6)	0.098		
Residual tumor	3.0 (0.9–8.3)	0.047		

Biomarkers	HR (95% CI)	*p*-value	Adjusted^#^ HR (95% CI)	*p*-value
AFP ≥ 400 ng/ml	2.7 (1.0–6.1)	0.031	3.5 (1.1–9.8)	0.025
AFP ≥ 20 ng/ml	1.8 (0.9–3.5)	0.100	1.6 (0.8–3.3)	0.181
AFP-L3 ≥ 10%	2.0 (1.0–3.9)	0.051	1.9 (0.9–3.8)	0.071
DCP ≥ 7.5 ng/ml	1.3 (0.6–2.6)	0.487	1.2 (0.6–2.5)	0.588
CTC status	2.5 (1.1–5.4)	0.025	2.7 (1.1–6.1)	0.019

Cox proportional hazards regression analysis for early recurrence free survival (eRFS) after resection, n = 49. #All Biomarkers were adjusted in a multivariate analysis for age, gender and BCLC stage.

*HR* hazard ratio, *CL* confidence interval.

For the detection of EpCAM-positive CTC, a hazard ratio of 2.5 (95% CI 1.1–5.4, *p* = 0.025) was calculated for eRFS. While an increase of AFP levels above the reference values did not correlate with a significantly worse eRFS (*p* = 0.100), a significantly worse eRFS could be demonstrated for the subgroup of patients with AFP levels ≥ 400 ng/ml (HR 2.7, 95% CI 1.0–6.1, *p* = 0.031). The remaining biomarkers, AFL-L3 (*p* = 0.051) and DCP (*p* = 0.487) did not reach statistical significance. In a multivariate analysis all biomarkers were adjusted for age, gender, and BCLC stage, a hazard ratio of 2.7 (95% CI 1.1–6.1, *p* = 0.019) was calculated for the detection of CTC and a hazard ratio of 3.5 (95% CI 1.1–9.8, *p* = 0.025) for AFP levels ≥ 400 ng/ml.

**Figure 2:**
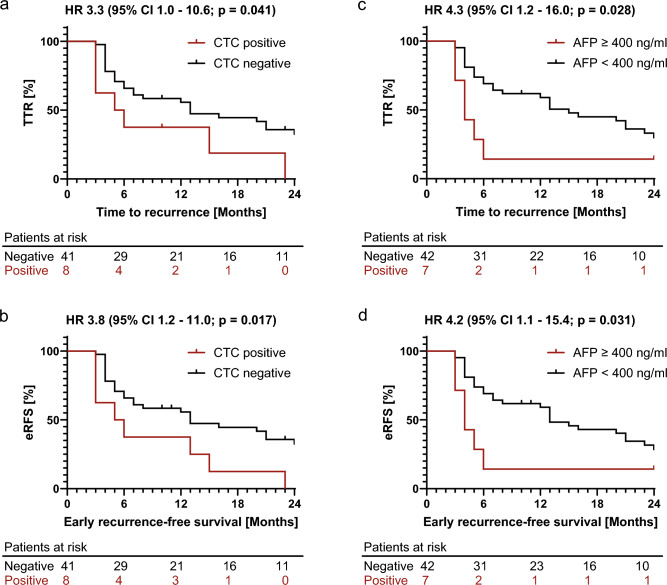
Early recurrence free survival and time to recurrence. Kaplan–Meier curves of (**a**) the time to recurrence (TTR) and (**b**) early recurrence-free survival (eRFS). TTR and eRFS is displayed according to CTC status (negative vs. positive) and AFP status (AFP ≥ 400 ng/ml vs. AFP < 400 ng/ml) (**c** and **d**). The *p*-value was calculated using the log-rank (Mantel-Cox) test. Abbreviations: HR, hazard ratio; CI, confidence interval.

We then divided patients into a CTC positive (n = 8) and CTC negative group (n = 41), a group with serum AFP levels ≥ 400 ng/ml (n = 7) and an AFP < 400 ng/ml group (n = 42). CTC and AFP levels ≥ 400 ng/ml were detected in two patients. eRFS and TTR were then plotted as Kaplan–Meier curves (Fig. 2). CTC positive status was associated with worse TTR (HR 3.3, 95% CI 1.0–10.6, *p* = 0.041) and eRFS (HR 3.8, 95% CI 1.2–11.0, *p* = 0.017). Median eRFS (5.5 vs. 13 months, *p* = 0.017) and median TTR (5.5 vs. 13 months, *p* = 0.041) were both significantly shorter in CTC positive patients. The specificity for recurrence was 94% (95% CI 0.72–0.99), with a sensitivity of 21% (95% CI 0.11–0.38). AFP levels ≥ 400 were associated with similarly poor TTR (HR 4.3, 95% CI 1.2–16.0, *p* = 0.028) and eRFS (HR 4.2, 95% CI 1.1–15.4, *p* = 0.031). Median eRFS (4 vs. 13 months, *p* = 0.031) and median TTR (4 vs. 15 months, *p* = 0.028) were both significantly shorter. The specificity for early recurrence was 94% (95% CI 0.72–0.99), with a sensitivity of 18% (95% CI 0.09–0.34).

### Combing CTC status and AFP levels as a composite biomarker

To evaluate CTC and AFP as a composite biomarker, patients were divided into a CTC positive or/and AFP ≥ 400 ng/ml group (n = 13) and a double negative group (n = 36). As described above, only two patients tested positive for CTC and AFP ≥ 400 ng/ml. There was no difference in patient characteristics such as age (*p* = 0.961), gender (*p* = 0.999), presence of cirrhosis (*p* = 0.753), Child–Pugh stage (*p* = 0.464), etiology of liver disease (*p* = 0.147), but a clear difference regarding tumor biology (Table 3). In the biomarker positive group, 61.5% of the patients showed vascular invasion in the histological workup of the resection, compared to 28.6% of the biomarker negative group (*p* = 0.049). Of note, there were no statistical differences for BCLC stage (*p* = 0.132) and positive resection margin (*p* = 0.999).

**Table 3:** Demographic characteristics of patients in regard of AFP levels and CTC status.

Baseline Characteristics	AFP ≥ 400 ng/ml or CTC positive (n = 13)	AFP < 400 ng/ml and CTC negative (n = 36)	*p*-value
Gender (%)			0.999
Male	11 (84.6)	31 (86.1)	
Female	2 (15.4)	5 (13.9)	
Age			0.961
Mean ± SD	63.7 ± 6.5	63.9 ± 11.9	
Liver cirrhosis (%)			0.753
Yes	7 (53.9)	17 (47.2)	
No	6 (46.2)	19 (52.8)	
Child–Pugh stage (%)			0.507
A	6 (85.7)	16 (94.1)	
B	1 (14.3)	1 (5.9)	
Etiology of liver disease#			0.147
Hepatitis B	3	5	
Hepatitis C	4	5	
NASH	1	8	
Alcohol	0	8	
Cryptogenic	0	4	
Multiple	2	2	
No liver disease	3	4	
BCLC staging (%)			0.132
A	8 (61.5)	30 (83.3)	
B	5 (38.5)	6 (16.7)	
Vascular invasion (%)*			0.049
V0	5 (38.5)	35 (71.4)	
V1/V2	8 (61.5)	10 (28.6)	
Residual tumor (%)			0.999
R0	12 (92.3)	33 (91.7)	
R1	1 (7.7)	3 (8.3)	
Biomarker			
CTC detected (Mean ± SD)**	1.1 ± 1.1	0	
AFP ng/ml (Mean ± SD)	1679 ± 2442	52 ± 87	
Outcome			
Median TTR	5 months	16 months	0.011
Median early RFS	5 months	16 months	0.005
Median OS	37 months	Not reached	0.034

*SD* standard deviation, *V* vascular invasion, *R* resection margin.

^#^Four patients had multiple risk factors and were combined as a distinct group for statistical analysis.

*No statement regarding vascular invasion was made by the pathologist for one resected specimen.

**Number of CTC detected with a minimum of 0 and a maximum of 3 cells detected.

**Figure 3:**
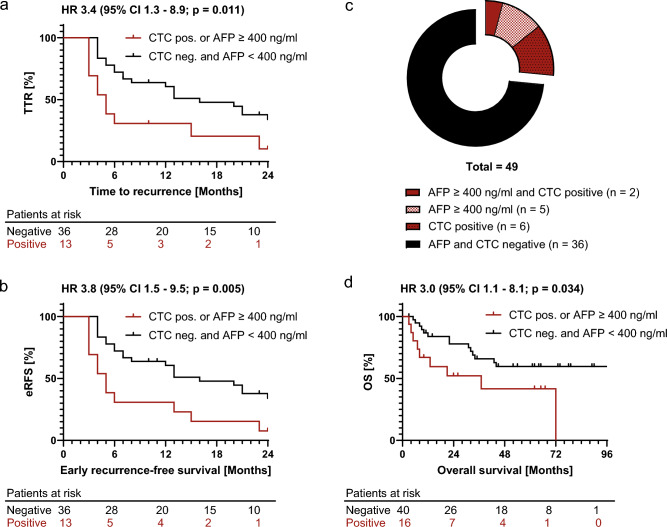
Early recurrence-free survival, time to recurrence and overall survival. Kaplan–Meier curves of (**a**) the time to recurrence (TTR), (**b**) early recurrence-free survival (eRFS) and (**d**) overall survival (OS). TTR, eRFS and OS is displayed according to combined CTC and AFP status (CTC positive or AFP ≥ 400 ng/ml vs. CTC negative and AFP < 400 ng/ml). The *p*-value was calculated using the log-rank (Mantel-Cox) test. (**c**) The distribution of biomarkers in the cohort with available recurrence data. For OS all patients were included regardless of available recurrence data (n = 56). Abbreviations: HR, hazard ratio; CI, confidence interval.

eRFS, TTR, and overall survival were plotted as Kaplan–Meier curves (Fig. 3). CTC positive status and/or AFP levels ≥ 400 ng/ml were associated with worse TTR (HR 3.4, 95% CI 1.3–8.9, *p* = 0.005) and eRFS (HR 3.8, 95% CI 1.5–9.5, *p* = 0.011). Median eRFS (5 vs. 16 months, p = 0.005) and median TTR (5 vs. 16 months, *p* = 0.011) were both significantly shorter. The specificity for early recurrence was 87% (95% CI 0.64–0.98), with a sensitivity of 33% (95% CI 0.20–0.50). Thus, the combination of both biomarkers increased sensitivity for the prediction of early recurrence while retaining a high degree of specificity.

A sub analysis of patients with cirrhosis and available recurrence data (n = 24) revealed a particularly poor eRFS (HR 8.0, 95% CI 2.0–31.7, *p* = 0.003) in the group positive for our composite biomarker (n = 7) compared to the double negative group (n = 17). Median eRFS was only 3 months in patients with cirrhosis and a positive composite test, whereas it was not reached in patients with a negative test (*p* = 0.003). For OS analysis all 56 available patients were included regardless of available recurrence data. CTC positive status and/or AFP levels ≥ 400 ng/ml were associated with worse OS (HR 3.0, 95% CI 1.1–8.1, *p* = 0.034). Median OS was 37 months for the group positive for our composite biomarker, while the median OS was not reached in the double negative group (*p* = 0.034).

## Discussion

The objective of this study was to evaluate a composite biomarker of EpCAM-positive circulating tumor cells and serum AFP to identify patients at high risk of early recurrence within 2 years after liver resection. While AFP is elevated in less than half of patients with HCC, it is associated with tumor aggressiveness and worse prognosis^9^. Detection of circulating EpCAM-positive CTC is considered a proxy for epithelial-mesenchymal transition (EMT), a key step in metastasis development. Both AFP and EpCAM-positive circulating tumor cells have previously been described to be highly specific tumor markers. However, the low sensitivity of both biomarkers alone limits their clinical utility^6,7,16,20^.

By univariate and multivariate Cox regression analysis, we confirmed previous studies indicating serum AFP levels ≥ 400 ng/ml and detection of EpCAM-positive CTC in peripheral blood prior to tumor resection as prognostic markers^7,16,28^. A statistical trend was observed for elevated AFP-L3 levels, albeit not statistically significant (*p* = 0.051). Of note, a recently published study was able to demonstrate prognostic value after microwave ablation for a combination of these three biomarkers^19^. For DCP positive patients (≥ 7.5 ng/ml) no significant difference could be detected (*p* = 0.487).

For patients with AFP levels ≥ 400 ng/ml, significantly shorter eRFS and TTR were observed. For recurrence, a high specificity of 94% was observed, with a sensitivity of 18%. Since in a majority of HCC cases AFP is not elevated in peripheral blood, this does not seem surprising. The selection of an appropriate cut-off value for AFP, therefore, remains a challenging task^29^. However, AFP levels ≥ 400 ng/ml are considered highly specific for poor oncological outcome after surgical resection^6–8^. Patients with CTC detection in peripheral blood also exhibited a significantly shorter eRFS and TTR, reported by us and others^16,28^. However, sensitivity is also low for CTCs.

We hypothesized that a combination of both biomarkers might constitute a highly specific test with improved sensitivity. In fact, only two patients tested positive for both CTC and AFP levels ≥ 400 ng/ml in our cohort, indicating complementary effects. Increased sensitivity would significantly enhance the utility of a composite biomarker. In the comparison of patients with CTC positive status and/or AFP levels ≥ 400 ng/ml and double negative patients, no differences in age, gender, etiology, cirrhosis status, or tumor stage were observed. However, a significant difference was seen with a higher percentage of vascular invasion in the resected specimens of the CTC/AFP ≥ 400 ng/ml group. Vascular invasion, as an indicator of aggressive tumor behavior, has already been described in the literature as a risk factor for recurrence^30^. A subsequent analysis of the primary endpoint eRFS showed a significantly shorter eRFS with 5 months versus 16 months (*p* = 0.005). There were also significant differences for the secondary endpoints TTR (*p* = 0.011) and OS (*p* = 0.034). The combined test continued to show a high specificity of 87% for recurrence while sensitivity improved significantly to 33%. We believe that this composite test will identify patients who are likely to have undetected micro-metastasis and could therefore represent the ideal target population for biomarker enriched trials testing (neo-)adjuvant therapies.

A recently published single-centre study of 73 patients with newly diagnosed HCC demonstrated that EpCAM-positive CTC and AFP levels ≥ 400 ng/ml are independent prognostic parameters for poor prognosis^31^. These results strengthen our hypothesis that the combination of AFP status and CTC are independent risk markers for patients with HCC. However, surgical resection was performed in only 5 of these patients, so no conclusion on recurrence can be drawn from this study. Another recent study demonstrated that a combination of AFP, AFL-L3, and CTC predicted recurrence after microwave ablation better than any single marker alone^19^. Interestingly, we also observed a similar trend for AFP-L3, although not statistically significant (*p* = 0.051). Finally, in a third study, AFP levels and CTC status were described as independent prognostic markers for recurrence after radiofrequency ablation^32^. All three publications strengthen our idea of AFP and CTC as independent biomarkers. In large multicenter studies, as well as in our cohort, AFP is elevated in only about half of all patients with HCC^9^. Since metastasis via CTC occurs independently of the AFP phenotype of the tumor, a combination of both markers seems particularly useful. To our knowledge, our study is the first study describing AFP and CTC as a composite test for recurrence prediction after surgical resection.

We believe that by combining both biomarkers, a group of patients with a very high risk of recurrence could be identified. This additional risk stratification seems particularly important considering current clinical trials in the adjuvant setting such as IMbrave050^33^. Here, the risk of recurrence was primarily determined by classical radiological and histological criteria such as tumor size or tumor grading, so only these patients were eligible for this trial^33^. Patients with a low risk of recurrence are currently not eligible for such studies. Therefore, the identification of patients at high risk for recurrence—independent of classical tumor features—appears to be of particular importance. Patients with positive CTC status or AFP ≥ 400 ng/ml appear to represent a high-risk population for recurrence in early-stage HCC, in whom adjuvant immunotherapy should be investigated. With the changing landscape of neoadjuvant immunotherapeutic approaches in other tumor entities, these composite biomarkers could also be used to identify patients with a high potential to benefit from neoadjuvant therapy. Conversely, a negative test would identify patients who might not need adjuvant therapy due to low risk of recurrence a priori.

Notably, in the subgroup of patients with cirrhosis a positive biomarker had a decisive impact on median eRFS (3 months vs. not reached, *p* = 0.003). These patients likely do not benefit from surgical resection alone. In this group, surgical risk factors should be weighed against the relatively low chance of recurrence-free survival.

The study design has inherent limitations and strengths that should be acknowledged in interpreting the results. The study was conducted in a single center and the sample size was relatively small, which may have limited the statistical power of the study. However, the primary and secondary endpoints of this study have been reached. The selection of patients for surgical resection was performed in our interdisciplinary tumor board, and if necessary, with prior evaluation for liver transplantation, to ensure the highest level of patient safety. Patient selection suitable for surgical resection might differ between surgical services. Notably, the study was conducted in a tertiary center with a representative Western population, and all etiologies were covered. Etiologies of HCC differ globally and data from a Western population might not be applicable to an Asian patient population where viral hepatitis is the dominant risk factor. To investigate the influence of different etiologies on CTC detection and AFP levels, a larger study group would be necessary. There was no validation cohort, which could have provided additional support for the findings. Therefore, previously described biomarker cutoff values were used to evaluate this study, which strengthens its validity even in the absence of a validation cohort.

Although not the aim of this study, previous work has shown that postoperative monitoring of CTC allows better characterization of the patient's risk of recurrence and this approach might be useful to further improve the results described in this study^34^. Further prospective studies are necessary to demonstrate the clinical utility of this approach. The detection of CTC via CSS was restricted to EpCAM and cytokeratin positive epithelial CTC. Different studies have suggested that newer technologies might lead to increased detection and characterization of CTC^35,36^. However, CSS has been established as a well-studied technique across different cancer entities including HCC^24,25^. Combination of this technology with well established biomarkers will facilitate further prospective studies on the topic for clinical implementation.

In summary, we demonstrated that by combining CTC status and AFP levels ≥ 400 ng/ml, a group of patients at high risk of postoperative tumor recurrence was prospectively identified. Especially for borderline cases and patients with increased intraoperative risk, this risk stratification seems reasonable. This biomarker combination might be useful to identify patients in very early tumor stages in whom additional adjuvant immunotherapies are indicated. To our knowledge, this represents the first combination of these biomarkers in a Western cohort after curative surgical resection.

## Data Availability

The datasets generated during the current study are available from the corresponding author on reasonable request after approval by the Ethics Committee of the Hamburg Medical Association.
